# Cyclic Adenosine Monophosphate Eliminates Sex Differences in Estradiol-Induced Elastin Production from Engineered Dermal Substitutes

**DOI:** 10.3390/ijms22126358

**Published:** 2021-06-14

**Authors:** Andreja Moset Zupan, Carolyn Nietupski, Stacey C. Schutte

**Affiliations:** 1 Department of Biomedical Engineering, University of Cincinnati, Cincinnati, OH 45221, USA; mosetzaa@ucmail.uc.edu (A.M.Z.); nietupca@mail.uc.edu (C.N.); 2 Department of Research, Shriners Hospitals for Children-Cincinnati, Cincinnati, OH 45221, USA

**Keywords:** elastin, dermal fibroblasts, estrogen, cAMP, tissue engineering

## Abstract

Lack of adult cells’ ability to produce sufficient amounts of elastin and assemble functional elastic fibers is an issue for creating skin substitutes that closely match native skin properties. The effects of female sex hormones, primarily estrogen, have been studied due to the known effects on elastin post-menopause, thus have primarily included older mostly female populations. In this study, we examined the effects of female sex hormones on the synthesis of elastin by female and male human dermal fibroblasts in engineered dermal substitutes. Differences between the sexes were observed with 17β-estradiol treatment alone stimulating elastin synthesis in female substitutes but not male. TGF-β levels were significantly higher in male dermal substitutes than female dermal substitutes and the levels did not change with 17β-estradiol treatment. The male dermal substitutes had a 1.5-fold increase in cAMP concentration in the presence of 17β-estradiol compared to no hormone controls, while cAMP concentrations remained constant in the female substitutes. When cAMP was added in addition to 17β-estradiol and progesterone in the culture medium, the sex differences were eliminated, and elastin synthesis was upregulated by 2-fold in both male and female dermal substitutes. These conditions alone did not result in functionally significant amounts of elastin or complete elastic fibers. The findings presented provide insights into differences between male and female cells in response to female sex steroid hormones and the involvement of the cAMP pathway in elastin synthesis. Further explorations into the signaling pathways may identify better targets to promote elastic fiber synthesis in skin substitutes.

## 1. Introduction

Mature elastic fibers are a key component of the dermis that is lacking in most skin substitutes and is reduced in aging skin. Elastin is essential to elastic fibers; it is an insoluble protein that makes up approximately 2–4% of the human dermal extracellular matrix [1,2]. It confers both mechanical and cell signaling properties upon tissues in which it is incorporated. As elastin has a half-life of approximately 70 years [3], cells past the early neonatal period generally do not produce elastin [4]. In the cases of injury when elastin is produced, the small amounts synthesized are insufficient to replace what was lost and the fibers are generally disorganized [5]. Loss of elasticity as part of the healthy aging process, [6,7] and disorders such as cutis laxa that occur due to defects in mature elastin [8,9,10,11], demonstrate the need for elastin in engineered skin substitutes to fully recapitulate normal healthy skin. While different methods have been attempted to stimulate elastin synthesis in the skin during wound healing or to incorporate elastin into scaffolds for engineered skin substitutes, none to date have been able to form a fully functional network of mature elastic fibers. This structural omission can lead to mobility and other quality-of-life issues following treatment; therefore, a better understanding of elastin synthesis is necessary to facilitate more favorable outcomes post-injury.

Estrogen is a regulator of elastin synthesis in the skin and other tissues as demonstrated by changes that occur post-menopause when estrogen levels drop [12,13,14,15]. The literature has already established that 17β-estradiol (E_2_), the most potent of the estrogens, can stimulate elastin synthesis in the skin by increasing transforming growth factor-β (TGF-β) [16,17,18]. Similarly, increases in elastin due to culture with E_2_ have been found in aortic smooth muscle [19] and adipose-derived mesenchymal stem cells [20]. Application of E_2_ also results in increased elastin content in the carotid artery [21], vagina [22], vocal fold [23,24], and skin [25]. Most of these studies utilized animal studies or human cells from aged participants. The conditions were often driven by trying to restore normal physiologic levels. While TGF-β has been identified as one mechanism that can lead to increased elastin synthesis, other mechanisms are likely. We hypothesized that cyclic adenosine monophosphate (cAMP) may also play a role. Studies have shown that cAMP does influence elastin synthesis; however, the results are limited and inconsistent [26,27] as elastin synthesis due to the addition of cAMP can vary based on concentration [28].

The current study was undertaken to investigate methods for increasing elastin synthesis in dermal substitutes, which may improve functional outcomes after transplantation to burn wounds or other wounds requiring skin grafts. Our goal was to have more generalizable data by studying the effects on both sexes in a large range of ages. The long-term goal is to identify methods to stimulate elastin synthesis in any adult dermal fibroblasts for skin graft and wound healing applications.

## 2. Results

### 2.1. Patient Demographics

Equal numbers of male and female patient samples were used in this study. There were no significant differences between the sexes in terms of age, race, ethnicity, or biopsy location. The patient demographics for the cells that were used are summarized in Table 1. The data below represents the demographics of the samples used for this study; where replicates were used, the samples were averaged and used as a single value for analysis.

### 2.2. Elastin Contents in Engineered Dermal Substitutes due to Steroid Hormone Culture

**Figure 1:**
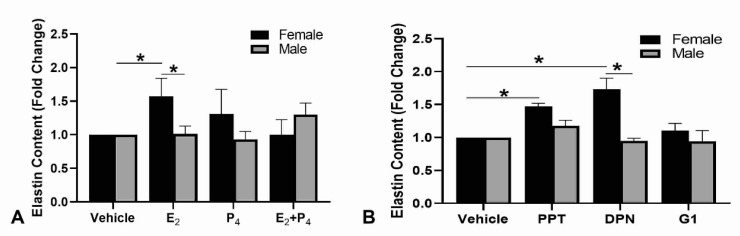
Elastin content of EDS. (**A**) Culture with E_2_ alone significantly increased elastin content in the female, but not the male EDS. (**B**) Culture with agonists specific for either ER-α (PPT) or ER-β (DPN) significantly increased elastin content in female EDS. Agonists to GPER-1 (G1) did not have a significant effect. n = 5, * indicates *p* < 0.05.

After culture, the elastin content of the tissues was measured and the results were normalized to DNA content. The fold changes in elastin content for treatment versus vehicle control were compared (Figure 1A). The elastin content was significantly increased after culture with E_2_ in the female engineered dermal substitutes (EDS) but not in the male EDS though the amounts of elastin in the EDS were still relatively low, 14.13 ± 2.88 mg/µg DNA in male EDS and 24.11 ± 4.91 mg/µg DNA in female EDS cultured with E_2_. To determine which receptors were involved in the increased elastin content after culture with E_2_, agonists specific to ER-α, ER-β, and GPER-1 were used in the culture medium of the EDS. Agonists for ER-α and ER-β led to significant increases in elastin in the female EDS only (Figure 1B). The amount of elastin after culture with the agonists was similar to that found after treatment with E_2_. This also confirms that the increase in elastin synthesis observed with the addition of E_2_ was indeed a function mediated via activation of the estrogen receptors.

### 2.3. Receptor Densities in the Human Dermal Fibroblasts

**Figure 2:**
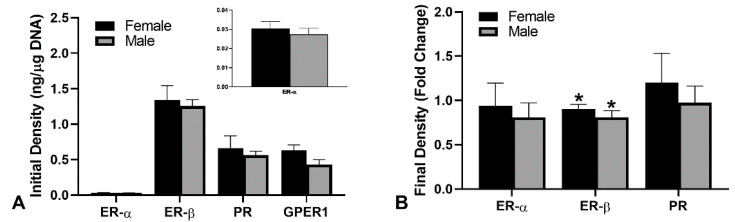
Receptor densities were similar in male and female cells but differ between receptor types. (**A**) initial receptor densities of the three estrogen receptors and the progesterone receptors. (**B**) After culture, the receptor densities were determined in the EDS homogenates and the fold change between vehicle control and either E_2_ treated (ERs) or P_4_ treated (PRs). n = 5; * indicates *p* < 0.05 compared to vehicle control.

The initial receptor densities were determined on isolated cells alone prior to being incorporated into the EDS to determine if differences in receptor number may be involved. No significant difference in receptor densities was observed between male and female human dermal fibroblasts (hDF) for any of the receptors (Figure 2). Final receptor densities were also quantified to determine if there were any changes to the receptor densities after culture with E_2_ or P_4_. Again, there were no significant differences in the quantity of ER-α after culture with E_2_ nor changes in the PR after culture with P_4_ in either the male or female EDS. In both cultures, the ER-β receptor densities significantly decreased in both male and female EDS after culture with E_2_ alone. This reduction was compared to vehicle controls which contained 6.20 ± 3.07 ng ER-β/µg DNA for male EDS and 6.09 ± 2.96 ng ER-β/µg DNA for female EDS.

### 2.4. Cellularity of the EDS

E_2_ is known to be mitogenic and as such could be a potential confounding factor when studying elastin content [29,30]. To ensure that the differences in the elastin content were due to elevated expression of elastin rather than increased cell number, the DNA content was determined for all EDS. The elastin content was normalized to DNA content before determining fold change. To look at overall cellularity, the DNA contents were normalized to protein content to account for any differences in tissue size. There were no significant changes in DNA content for any conditions when evaluated as content alone or as fold change compared to vehicle control. The female EDS and male EDS cultured with the vehicle control contained 3.51 ± 1.67 and 3.86 ± 1.31 µg DNA/mg protein content, respectively. When cultured with E_2_, the contents were 3.56 ± 1.18 and 3.72 ± 0.74 µg DNA/mg protein content, which represents a change of 1.01 ± 0.11-fold and 1.03 ± 0.25-fold compared to the vehicle for female and male EDS, respectively.

### 2.5. TGF-β1 and cAMP Concentrations in the Culture Medium after Culture with E_2_

**Figure 3:**
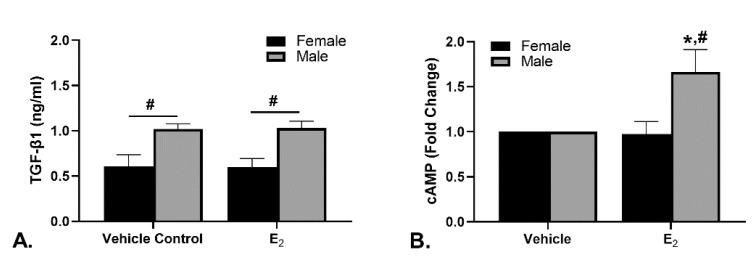
TGF-β1 and cAMP concentrations after E_2_ culture. (**A**) The concentration of TGF-β1 was higher in male cultures than in female cultures but was not affected by treatment. (**B**) The presence of cAMP in the male tissues treated with E_2_ was significantly higher than that of females and the vehicle control. n = 5; * indicates *p* < 0.05 compared to vehicle control; # indicates *p* < 0.05 compared to the opposite sex.

The TGF-β1 concentration in the spent medium was investigated as a possible mechanism for the differences in elastin synthesis (Figure 3A). There were no significant changes in the TGF-β1 concentration due to E_2_ treatment when compared to control for either the female or male EDS. There was a significant difference between the sexes with the male EDS producing significantly more TGF-β1 than the females which does not correspond with the changes in elastin synthesis.

The cAMP levels in the EDS homogenates were determined (Figure 3B). These data are presented as the fold change of the cAMP concentration normalized to DNA content. The male EDS homogenates had significantly increased cAMP levels in response to E_2_ compared to vehicle and the female EDS. The average concentration of the vehicle control for male EDS was 2.66 ± 0.54 fmol/µg DNA and the average for the female EDS was 2.41 ± 0.26 fmol/µg DNA.

### 2.6. Elastin Content When Exposed to Steroid Hormones and cAMP

**Figure 4:**
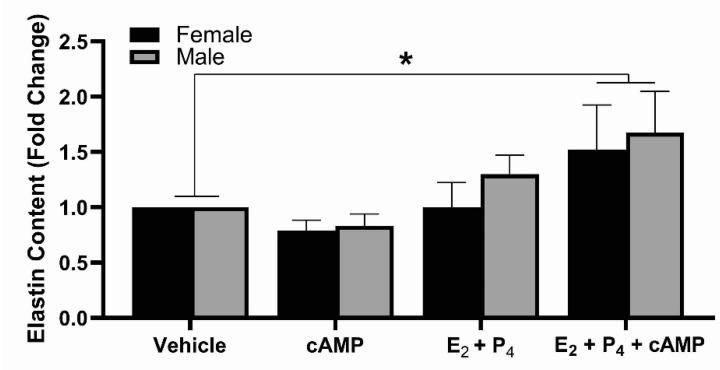
Effects of cAMP on elastin synthesis. The addition of cAMP alone does not affect elastin synthesis; however, when added with E_2_ and P_4_ an increase in elastin synthesis in both male and female EDS occurs. n = 5; * indicates *p* < 0.05.

The addition of cAMP alone did not lead to an increase in elastin synthesis; however, when combined with E_2_ and P_4_ elastin synthesis significantly increased in both male and female EDS (Figure 4).

## 3. Discussion

This study has confirmed that E_2_ has a stimulatory effect on elastin production in EDS containing female hDF, and further shows this is via activation of the nuclear ERs, primarily ER-β. The elastogenic effect of E_2_ treatment was absent in male EDS and cAMP levels in the EDS in response to E_2_ were significantly higher in the male EDS than in the female or vehicle. The sex differences in elastin synthesis could be eliminated when the EDS were cultured with cAMP, E_2,_ and P_4_. The elastogenic effects of E_2_ on hDF have been previously demonstrated in vivo and in vitro [16,18,31]. Several of these studies have also found correlations between TGF-β1 levels and the increased elastin synthesis in response to E_2_ [16,32]. Unlike these studies, TGF-β1 levels were not altered in our EDS cultures in response to E_2_, suggesting other pathways are involved.

The effects of sex hormones on gene expression are dependent on the presence of sex steroid hormone receptors within the tissue. It is now well recognized that the actions of estrogen are mediated via interaction with the two nuclear estrogen receptors (ERα and ERβ) which function as transcription factors within the nucleus, and/or the membrane estrogen receptor (GPER-1), which activates specific second messenger signaling pathways. Due to the different molecular mechanisms by which estrogen action is modulated upon activation of these receptors, estrogen signaling can be classified as genomic (ER-α/β) or non-genomic (GPER-1). Similar to the ERs, the progesterone receptor functions as a classic nuclear receptor and mediates gene expression via genomic signaling when bound to progesterone.

The male and female EDS were shown to express the same initial density of hormone receptors; however, their level of activity in response to the same treatment conditions with respective hormones/agonists was dramatically different between male and female EDS. Our findings indicate that treatment with E_2_ increases elastin content selectively in females. A similar effect could be achieved with ER selective agonists, confirming that the increase in elastin synthesis was a result of increased activation of the ERs in response to E_2_ in females. When the GPER-1 selective agonist was used, no change in elastin for either female or male EDS was observed. Because the primary effect of GPER activation is stimulation of adenylyl cyclase, which subsequently catalyzes the production of cAMP, we next sought to determine if cAMP levels were altered in EDS in the presence of E_2_. In contrast to the ERs, we found that cAMP levels were significantly increased in response to E_2_ in the male EDS, but unaltered in the female compared to vehicle. This indicates that although E_2_ treatment did not lead to increased elastin production, E_2_ did induce signaling via nongenomic mechanisms in males.

Collectively, these findings demonstrate sex-specific differences in signaling mechanisms in which E_2_ signals via the nuclear ERs selectively in females, and we hypothesize that it signals via GPER-1 selectively in males. The theory that estrogen can function as a sex-specific biased agonist presents a novel and sophisticated mechanism by which hormones may regulate gene expression, and specifically in the current study, underly the difference in elastin expression observed between the female and male EDS. Furthermore, the differential outcome in elastin synthesis between male and female EDS following culture with E_2_ could be attributed to the relative density levels of the GPER and the ERs. While the density of hormone receptors did not differ between sexes, there were significant differences in the density levels among the receptor types. If E_2_ selectively signals through ERs in females and GPER-1 in males, then the higher receptor density, and thus signal response, would support why E_2_ alone could increase elastin in females only.

While culture with E_2_ or P_4_ alone did not alter elastin content in male EDS, there was a non-significant, but observable increase in elastin when cultured in presence of both E_2_ and P_4_ in the male cells. One explanation for this could be an increase in GPER density, thus signaling, in response to P_4_. We did not measure receptor levels following coculture with E_2_ and P_4_, but this notion is supported by results from previous studies that demonstrate progestin up-regulates GPER-1 expression [33]. This P_4_-induced increase in GPER-1 expression paralleled an increase in receptor-bound estrogen, which also correlated to increased cAMP production [34]. When exogenous cAMP was also added to culture treatment with E_2_ and P_4_, a further increase in elastin was seen in males, resulting in a significant increase in elastin content compared to vehicle in both male and female EDS. Co-expression of ER-α and/or ER-β with GPER-1 suggests the possibility of interactions between these receptors and likely involves cross-talk between their signaling pathways. The observation that the addition of P_4_ with E_2_ treatment increased elastin while also eliminating the E_2_-induced increase elastin via ER activation in females raises the possibility of coordinated hormonal control of GPER-1 and the ERs by P_4_ in hDF. We believe that this increase in elastin synthesis is likely due to upregulation of GPER-1 in males, and that this response will also be present in other cells expressing these receptors. By estrogen acting as a GPER-1-biased ligand in males, a trend of increasing elastin content could be proposed from the collective findings presented here.

While there was an increase in elastin content in our EDS, no elastic fibers were found and the overall production of elastin was still low. Because this was a relatively short-term study, a longer culture may lead to different results, as elastic fibers are not seen in wound repair until weeks to months after injury [35,36,37]. We believe that these results are still significant, as tropoelastin synthesis, the pro-form of elastin, is a limiting factor in elastic fiber production in engineered skin [38]. Additionally, we have discovered a proposed mechanism for sex-specific signaling and a method by which these sex differences could be eliminated. By further investigating the pathways involved, new methods to stimulate the production of elastin by hDF in skin substitutes may be found. Mechanical forces have also been shown to induce elastin synthesis and result in elastic fiber formation in blood vessel substitutes. The combination of mechanical forces and hormones may result in elastic fiber formation and a better understanding of the specific mechanisms involved.

A major limitation of this study is the sample size along with the presence of confounding factors. The confounding factors of sex, age, and race in combination with the sample size increased the sample variability. Age is known to have an impact on elastin synthesis, in response to E_2_ treatment older hDF synthesize more elastin than younger hDF, especially in females [16,37]. Race is also known to influence elastin synthesis; Black subjects were found to produce more TGF-β and, thus, elastin than their White counterparts [39]. This study aimed to find more generalizable data; however, these factors need to be considered. A larger sample size may be needed or, if it can be justified, a smaller range of ages in subjects should be considered.

This study demonstrated that there are culture conditions by which we can induce elastin synthesis in both male and female hDF. This is a first step towards determining methods for stimulating elastin synthesis and assembly by hDF used in fabrication of engineered tissue substitutes. By identifying pathways, additional targets can also be identified for wound healing applications, where drugs can be developed and applied topically or through drug eluting scaffolds to promote proper elastin synthesis and assembly.

## 4. Materials and Methods

### 4.1. Tissue Sources

De-identified, discarded human skin was obtained from elective plastic and reconstructive surgery procedures at the University of Cincinnati Medical Center and Shriners Hospitals for Children—Cincinnati.

### 4.2. Isolation and Culture of Primary Dermal Fibroblasts

Isolation of hDF was performed following the method described in McFarland et al. [40]. Briefly, the full-thickness skin was incubated overnight at 4 °C in Dispase II (Sigma-Aldrich, St. Louis, MO, USA), after which the epidermis was mechanically separated from the dermis. The dermis was minced and digested with type II collagenase (Worthington, Lakewood, NJ, USA), with periodic agitation. Cells were cultured with medium consisting of Dulbecco’s modified eagle medium (DMEM, Gibco, Grand Island, NY, USA) supplemented with 5% *v/v* fetal bovine serum (FBS, Gibco), 10 ng/mL epidermal growth factor (Peprotech, Rocky Hill, NJ, USA), 0.5 μg/mL hydrocortisone (Sigma), 5 μg/mL insulin (Sigma), 0.1 M L-ascorbic acid 2-phosphate sesquimagnesium salt hydrate (AA2P, Sigma), and 1% *v*/*v* antibiotic-antimycotic (Gibco). The hDF were expanded and utilized between passages 1 and 3. The methods described for isolation have been used extensively and results in >98% purity of the dermal fibroblasts; identification for this study was primarily based on morphology. Cells from each batch are archived.

### 4.3. Fabrication and Culture of Engineered Dermal Substitutes

EDS were fabricated by mixing hDF with fibrinogen (Sigma), 25 U/mL thrombin (Sigma), and tissue culture medium for a final concentration of 2 × 10^6^ cells/mL and 2 mg/mL fibrin. After fibrillogenesis, additional tissue culture medium consisting of DMEM supplemented with 10% FBS, 1% antibiotic-antimycotic, 2 mg/mL ε-amino-η-caproic acid (ACA, EMD Millipore, Burlington, MA, USA), and 50 μg/mL AA2P was added. ACA inhibits plasminogen activation and thereby inhibits fibrin degradation. Over the first 2 weeks, the ACA content of the medium was gradually reduced to a final concentration of 1.25 mg/mL at which time the EDS were rinsed in phosphate-buffered saline (Sigma) and placed in hormone-containing medium for an additional 2 weeks. The hormone medium consisted of phenol red-free DMEM (Gibco) supplemented with 10% charcoal stripped-FBS (Innovative Research, Novi, MI, USA), 1% antibiotic-antimycotic, 1.25 mg/mL ACA, 50 μg/mL AA2P, and the desired hormones, agonists, or appropriate vehicle control. The steroid hormones E_2_ (Sigma) and P_4_ (Sigma) were solubilized in 10% ethanol (Sigma) and used alone or in combination at concentrations of 10 nM and 100 nM, respectively. Dibutyryl cAMP (Sigma) was used at a concentration of 500 μM. To determine which estrogen receptors were involved, 10 μM propyl pyrazole triol (PPT, Tocris Bioscience, Minneapolis, MN) and 10 μM diaproprionitrile (DPN, Tocris), or 1 µM G1 (Cayman Chemicals, Ann Arbor, MI, USA) were used. These are agonists for ER-α, ER-β, and GPER-1 respectively. The ER agonists were solubilized in dimethyl sulfoxide (Sigma) which was used as the vehicle control for the agonist studies. The EDS were cultured for an additional 2 weeks with hormones or agonists.

### 4.4. Protein and DNA Quantification

ELISAs were used to quantify the concentrations of ER- α, ER-β, GPER-1, and the progesterone receptors (all from Aviva Systems Biology, San Diego, CA, USA) and cAMP (Enzo Life Sciences, Inc., Farmingdale, NY, USA) in the tissue homogenates. The spent medium was assayed using an ELISA for TGF-β1 (R&D Systems, Minneapolis, MN, USA). The Fastin™ Elastin assay kit (Accurate Chemical, Westbury, NY, USA) was used for quantitation of elastin in the tissue per manufacturer instructions. The tissue samples for the elastin assay were frozen and stored at −80 °C prior to analysis. For total protein quantification and ELISAs, the samples were placed in RIPA buffer (Sigma) with proteinase inhibitor (Sigma) and homogenized. Protein quantification was performed via the Pierce BCA protein assay kit (ThermoFisher Scientific, Rockford, IL, USA). DNA content was determined for cell lysates and tissue homogenates using a Hoescht 33258 dye (Sigma) assay described previously [41]; calf thymus DNA (Sigma) was used for a standard curve. This was used for direct comparisons as well as for normalization.

### 4.5. Statistical Analysis

Data are presented as mean ± standard error of the mean. Statistical analysis was performed using JMP Pro 15 (SAS Institute, Inc., Cory, NC, USA). Student’s *t*-tests were used for comparison of age and initial receptor densities. Chi-squared analysis was used to compare race. A two-way ANOVA with a post hoc Tukey analysis was performed for the analysis of elastin, collagen, receptor density, and DNA contents. Statistical significance was determined using a 95% confidence interval.

## Figures and Tables

**Table 1:** Sample source demographics.

	Females	Males	*p*-Value
**Age (yrs)**	28.4 ± 17.4	22.1 ± 1.9	0.26
**Race**			0.58
White	2	3	
Black	3	2	
**Ethnicity**			0.93
Hispanic	1	0	
Non-Hispanic/Not disclosed	4	5	
**Biopsy Location**			0.17
Abdomen	1	3	
Breast	3	0	
Foot	0	1	
Thigh	1	1	

## Data Availability

The data presented in this study are available on request from the corresponding author.
